# Simplified Knee MRI ‘Sagittal Tibial Epi-Physis (STEP)’ Shorthand for Skeletal Age Assessment in Pediatric Patients with ACL Injury

**DOI:** 10.3390/diagnostics16030442

**Published:** 2026-02-01

**Authors:** Alberto Grassi, Claudio Rossi, Luca Ambrosini, Yuta Nakanishi, Emre Anil Ozbek, Amir Assaf, Hikaru Kayano, Mohammad Ibra Alhalalmeh, Kyle Borque, Stefano Zaffagnini

**Affiliations:** 1 II Orthopedics and Trauma Unit, IRCCS Istituto Ortopedico Rizzoli, Via Giulio Cesare Pupilli 1, 40136 Bologna, Italyluca.ambrosini3@studio.unibo.it (L.A.); 11.6.88a@gmail.com (A.A.); drmohmadhalalmeh@gmail.com (M.I.A.); stefano.zaffagnini@unibo.it (S.Z.); 2 Bioengineering and Computing Laboratory, (DIBINEM), University of Bologna, 40136 Bologna, Italy; 3 Department of Orthopaedic Surgery, Kobe University Graduate School of Medicine, Kobe 650-0017, Hyogo, Japan; y.n.fuzuki18@gmail.com; 4 Department of Orthopedics and Traumatology, Ankara University, 06000 Ankara, Turkey; ozbekanilmd@gmail.com; 5 Medical Faculty, Osaka University, Suita 565-0871, Osaka, Japan; hikaru.kayano.0257@gmail.com; 6 Department of Orthopaedic Surgery, Houston Methodist Hospital, 6550 Fannin St, Houston, TX 77030, USA; kaborque@gmail.com

**Keywords:** skeletally immature, physeal sparing, ACL, skeletal age, MRI, bone age, shorthand, atlas

## Abstract

**Objectives:** To develop a simplified MRI-based shorthand assessment method, referred to as the Sagittal Tibial Epi-Physis (STEP) Shorthand, for skeletal age assessment in skeletally immature patients with anterior cruciate ligament (ACL) injuries. This study aimed to elaborate a single-plane MRI-based skeletal age estimation tool and to explore its feasibility and inter-rater reliability in comparison with existing MRI-based shorthands. **Methods**: This prospective study included 130 knee MRIs (79% males) from 97 skeletally immature patients (overall average age of 14.0 ± 2.1 years) with ACL injuries treated between February 2022 and January 2025. A new shorthand assessment method was developed based on sagittal T1-weighted MRI evaluation of the proximal tibial epiphysis. A validation cohort of 74 MRIs was independently evaluated by four raters with different levels of expertise using the STEP, Meza, and Politzer shorthand atlases. Inter-rater reliability (ICC), intra-rater agreement (Cohen’s kappa), and association with chronological age (Spearman rho) were calculated. **Results**: The STEP Shorthand tool demonstrated a strong association with chronological age (rho = 0.890, *p* < 0.001) with consistent associations across sex subgroups. Inter-rater reliability was high and comparable to established MRI-based shorthands. The use of a focused sagittal T1-weighted evaluation allowed for a simplified and reproducible assessment across raters with varying experience levels. **Conclusions**: The STEP Shorthand represents a pragmatic and reliable tool for MRI-based skeletal age assessment in pediatric and adolescent patients with ACL injuries. The STEP Shorthand can support timely decision-making in surgical planning and enhance standardization across different levels of clinical expertise.

## 1. Introduction

Anterior cruciate ligament (ACL) reconstruction in skeletally immature patients requires careful surgical planning to avoid growth plate damage and subsequent growth disturbances [1]. Surgical decision-making between physeal sparing and transphyseal reconstruction techniques primarily relies on accurate skeletal age assessment [2].

Traditionally, skeletal maturity evaluation has depended on hand and wrist radiographs, with the Greulich and Pyle (G&P) atlas widely accepted as the gold standard [3]. However, three significant limitations emerge when applying this method to pediatric knee pathologies: (1) assessment occurs at a site anatomically remote from the knee; (2) correlation with knee-specific skeletal development remains uncertain; and (3) the technique requires additional radiation exposure and increases the costs [4].

To overcome these limitations, the development of MRI-based skeletal age assessment at the knee has provided a joint-specific, radiation-free alternative [5]. In 2018, Pennock et al. developed and validated an MRI-based knee bone age atlas, improving skeletal maturity assessment in pediatric patients, particularly those presenting with knee pathology. The atlas, derived from over 850 MRI scans, captures a reproducible sequence of ossification changes in the femur, tibia, fibula, and patella, and demonstrated excellent intra- and interobserver reliability. Notably, in a subset of patients who underwent both hand radiography and knee MRI, the atlas showed a strong correlation with the Greulich and Pyle (G&P) method, supporting its use as a reliable alternative to the traditional hand-based assessment. [6]. Based on this work, other authors have proposed their “shorthand” methods to facilitate knee MRI interpretation in clinical practice, such as Meza et al. [7] and Politzer et al. [8]; nevertheless, their use in clinical practice can be challenging as they require evaluation of various anatomical structures across multiple imaging planes, thus potentially affecting both practicality and interobserver reliability.

To address these limitations, the Sagittal Tibial Epi-Physis (STEP) Shorthand was developed as a novel simplified MRI-based method focusing specifically on proximal tibial physis morphology. The present study aimed to validate the STEP Shorthand method for skeletal age estimation in skeletally immature patients with ACL injuries, comparing its reliability characteristics with existing MRI-based atlases.

## 2. Methods

All skeletally immature patients with ACL rupture treated at the Rizzoli Orthopaedic Institute (Bologna, Italy) between February 2022 and January 2025 by a single surgeon (A.G.) were prospectively enrolled in this study. Diagnosis of ACL rupture was based on medical history, clinical examination, and MRI assessment. Patients were considered “Skeletally Immature” if either the tibial or femoral physis was open on their knee MRI. The exclusion criteria were multi-ligament injuries, revision ACL procedures, patients without postoperative MRI, and patients with closed physes. Patients were enrolled whether they were treated surgically or non-operatively.

### 2.1. Creation of the Sagittal Tibial Epi-Physis (STEP) Shorthand

From the findings of the skeletal age MRI knee atlas developed by Pennock et al. [6] a new stepwise algorithm for predicted knee MRI bone age was developed separately for male and female patients. The Pennock Atlas is based on the timing of ossification of patella, tibia, femur, fibula and physeal closure, evaluated on sagittal and coronal views. Since it includes the assessment of different anatomical segments on different views, a new original and simplified algorithm was developed including only the assessment of the proximal tibial epiphysis on the sagittal T1 view.

Briefly, the MRI characteristics of “Tibial tubercle extension of the epiphysis”, “Presence of tibial tubercle apophyseal center” and “Presence of tibial tubercle ‘crack’” were maintained from the original Pennock atlas. The “Presence of the Oreo Sign” which is assessed on the femoral condyles was substituted with the feature “Tibial continuous cartilage, >1.5 mm, not multilaminar”. The latter feature was demonstrated by Ekizoglu et al. to be present in male patients with a median age of 14.3 years, similar to the age milestone of the “presence of the Oreo Sign” in the Pennock atlas (14 years). Similarly, the Pennock feature “Femoral Oreo sign complete disappearance” was substituted with “Tibial continuous cartilage, >1.5 mm, not multilaminar”, which anticipates the closure of tibial cartilage [9].

However, the presence and absence of the *“Oreo Sign”* was maintained as a secondary feature in the present shorthand for skeletal age confirmation. The feature *“Tibial physis central closure”* was maintained from the Pennock atlas and modified only by adding *“…with femoral physis fully open”*. The Pennock feature *“Femoral physis central closure”* was modified in *“Tibial physis anterior and central closure with Femoral physis central closure”*, while the feature *“Tibial physis complete closure”* was modified in *“Tibial physis complete closure with Femoral physis anterior and central closure”.* When assessing femoral and tibial closure, the assessment on coronal slices was considered as a secondary finding to improve reliability.

The following characteristics were thus included in the STEP Shorthand:

- *Tibial apophysis without extension of the epiphysis*: assessed on the sagittal T1 images, it is present when the ossification of the proximal tibial epiphysis is not extending downward toward the tibial tubercle.
- *Tibial apophysis with extension of the epiphysis*: assessed on the sagittal T1 images, it is present when the ossification of the proximal tibial epiphysis is extending downward toward the tibial tubercle; however, the ossification center of the tibial tuberosity itself is not present yet.
- *Tibia apophysis ossification center:* Assessed on the sagittal T1 images, it is identified when a discrete ossification nucleus inferior to the tibial physis is present. It indicates that the tibial tubercle is starting to develop, and that the maturation of the proximal epiphysis is almost complete.
- *Tibial apophysis partial fusion “crack”*: Assessed on the sagittal T1 images, it is identified as a thin hypointense line separating the tibial epiphysis and the tibial tubercle apophysis. It indicates that the two structures are near to complete fusion between with each other.
- *Tibial continuous cartilage, >1.5 mm, not multilaminar*: Assessed on the sagittal T1 images, it is identified as a continuous horizontal linear cartilage signal intensity present between the tibial metaphysis and the epiphysis, with a thickness greater than 1.5 mm, with increased signal intensity but without a multilaminar appearance. The “crack” sign is not present. It indicates that the tibial apophysis is recently fully ossified.
- *Tibial continuous cartilage, <1.5 mm, not multilaminar*: Assessed on the sagittal T1 images, it is identified as a continuous horizontal linear cartilage signal intensity present between the metaphysis and the epiphysis, with a thickness less than 1.5 mm. It indicates that tibial growth cartilage is still open but is about to start the closing process.
- *Tibial physis central closure, femoral physis fully present*: Evaluated on coronal T1 images, the course of tibial growth cartilage is assessed from the peripheral portion to the center of the bone; in this case, the center of the growth cartilage is fused, while the anterior and posterior portions are still visible. The femoral growth cartilage is instead completely visible and has not started the ossification process yet.
- *Tibial physis anterior and central closure, femoral physis central closure*: Evaluated on coronal T1 images, the course of growth cartilage is assessed from the peripheral portion to the center of the bone; in this case, the tibial growth cartilage is fused from the anterior to the central portion, while it remains visible only in the posterior portion. The femoral growth cartilage of the femur is fused only in its central portion.
- *Tibial physis complete closure, Femoral physis anterior and central closure*: Evaluated on coronal T1 images, the course of growth cartilage is assessed from the peripheral portion to the center of the bone; in this case, the tibial growth cartilage is completely fused from the anterior to the posterior portion. Likewise, the femoral growth cartilage of the femur is almost completely fused only in its anterior and central portion.

Other secondary signs are included in the STEP Shorthand to help in the assessment of the correct skeletal age, in doubtful cases:

- *“Oreo” sign*: Assessed on the sagittal T1 images at the level of the femoral condyles, it is defined as a laminated appearance of the subchondral epiphyseal cartilage, usually as black-grey-black layers. When the subchondral cartilage presents as a single black layer, the “Oreo” sign is considered to be disappeared.
- *Tibial and Femoral growth cartilage in coronal views*: When assessing the status of growth cartilage on sagittal slices, its complete or partial closure could be confirmed on the T1 coronal view. Considering the larger size of the tibia and femur in coronal view, the status of the growth cartilage can be better observed, especially in the most posterior cuts.

**Figure 1:**
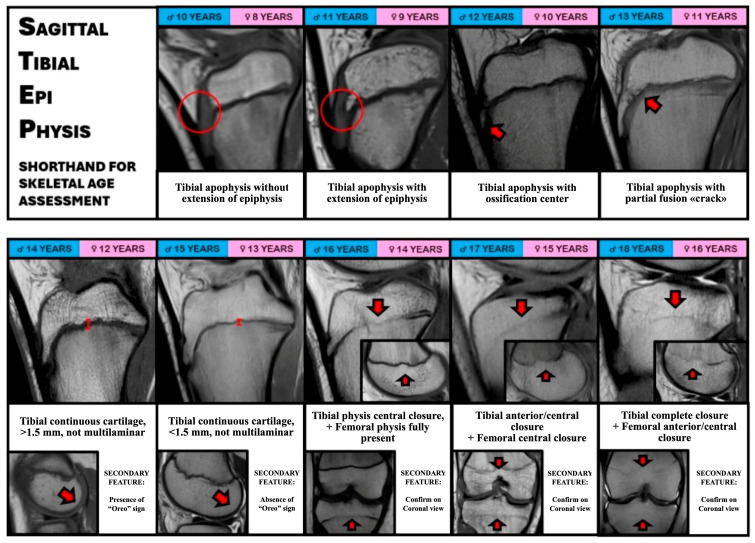
STEP (Sagittal Tibial Epiphysis) Shorthand for skeletal age assessment. Representative sagittal T1-weighted MR images of the tibial epiphysis and apophysis across different chronological ages in male (blue) and female (pink) patients. The progression of tibial apophyseal development, ossification center appearance, and subsequent physeal closure is illustrated, with key landmarks indicated by red arrows. Secondary features, including the “Oreo sign” and confirmation of physeal closure on coronal views, support accurate assessment in equivocal cases.

Finally, based on the former characteristics, the STEP Shorthand was created (Figure 1).

### 2.2. Application of the Sagittal Tibial Epi-Physis (STEP) Shorthand

For the development of the STEP Shorthand, a cohort of 97 skeletally immature patients (130 knee MRIs) was analyzed. A single surgeon with more than 8 years of experience in ACL reconstruction in adult and pediatric patients reviewed all the MRIs in order to identify consistent MRI features, based on existing MRI-based shorthands, to inform the development of the present shorthand. The median age of the patients included in each age milestone was calculated and divided according to male or female sex. Skeletal age was also compared with chronological age, and the difference was calculated for the complete cohort and for the male and female subgroups.

### 2.3. Validation of the Sagittal Tibial Epi-Physis (STEP) Shorthand

For validation purposes, an independent subset of 74 MRIs was selected from the overall cohort. Four examiners with different levels of expertise (1 medical student, MS; 1 orthopedic resident at PGY-3, OR; 1 orthopedic surgeon, OS; 1 fellowship-trained orthopedic surgeon with 1 year of training in pediatric ACL reconstruction, FT) evaluated these MRIs and assessed skeletal age according to the Meza et al. [7], Politzer et al. [8], and STEP shorthands. All raters performed independent evaluations and were blinded to chronological age, sex, clinical information, and surgical timing. MRI examinations were anonymized and presented in randomized order. The Interclass Correlation Coefficient (ICC) among the 4 different examiners was calculated for the three shorthands, while the inter-rater agreement between each single examiner was calculated for each shorthand. Eventually, the correlation coefficient was calculated between the chronological age and each value of skeletal age from all raters and all shorthands.

### 2.4. Statistical Analysis

Statistical analysis was performed with MedCalc (MedCalc Software Version 23.2.1, Acacialaan, 22 Ostend, Belgium). Continuous variables were reported as mean ± standard deviation, while categorical variables were reported as absolute number and proportion of the total sample. When multiple variables were compared, the one-way ANOVA test was used. The Spearman rho (rS) was used to correlate the mean knee MRI bone age scores from all raters and among different shorthands; correlation was classified as negligible (0.00–0.10), weak (0.10–0.39), moderate (0.40–0.69), strong (0.70–0.89) or very strong (0.90–1.00) [10].

The predicted bone ages for each individual rater were compared across readings using a 2-way, mixed effects, absolute agreement model of ICC. For all ICC comparisons, the following cut offs were used: poor (0.00–0.39), fair (0.40–0.59), good (0.60–0.74), and excellent (0.75–1.00) [7,8,11].

The inter-rater reliability was assessed with the Cohen’s kappa, and interpreted according to the following cut-offs for agreement: poor (0.00–0.20), minimal (0.21–0.39), weak (0.40–0.59), moderate (0.60–0.79), strong (0.80–0.90), and almost perfect (>0.90) [12]. Statistical significance was set at *p* < 0.05.

## 3. Results

### 3.1. Patient Characteristics

A total of 130 MRIs from 97 patients (75 males and 22 females) with ACL injury and open physis were assessed. A total of 23 patients had two consecutive MRIs and 5 patients had three consecutive MRIs with an interval of at least 6 months between the two exams. The overall average age was 14.0 ± 2.1 years, with male patients having an average age of 14.0 ± 2.2 years and female patients of 13.9 ± 1.5 years. No significant differences were present between the two genders (*p* > 0.05).

### 3.2. Sagittal Tibial Epi-Physis (STEP) Shorthand Characteristics

At least five MRIs were present for each bone age from <11 years to 18 years for male patients, while no MRI with a skeletal age of <9 years to 11 years and only one with a skeletal age of 12 years were present for female patients (Table 1). Thus, no female patients with incomplete tibial apophysis ossification were present in this series.

The median age of the patients included in each age group had a difference of less than 6 months compared to the specific age milestone, demonstrating an acceptable capacity of the shorthand to discriminate the different age groups (Table 2).

Moreover, there were no significant differences between chronological age and skeletal age according to the shorthand either for the whole cohort (14.0 ± 2.1 vs. 14.1 ± 2.3; *p* > 0.05) or for the subgroups of male patients (14.0 ± 2.2 vs. 14.0 ± 2.6; *p* > 0.05) and female patients (13.9 ± 1.5 vs. 14.1 ± 1.6; *p* > 0.05). A strong correlation was found between chronological age and skeletal age according to the STEP Shorthand (rho = 0.890; 95% CI 0.833–0.929; *p* < 0.001).

### 3.3. Sagittal Tibial Epi-Physis (STEP) Shorthand Reliability and Comparison with Other Shorthands

There were no significant differences between the mean skeletal age measured by the four raters for the present shorthand, the Politzer et al. [8] shorthand, and the Meza et al. [7] shorthand (Table 3).

**Figure 2:**
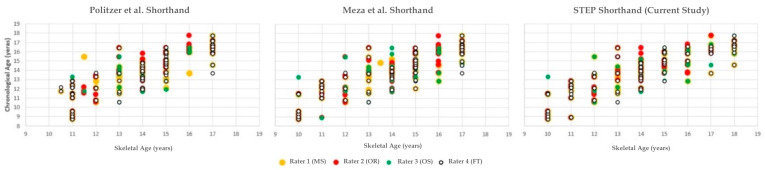
Scatter plots show chronological age versus skeletal age as assessed with the Politzer Shorthand (**left**), the Meza Shorthand (**middle**), and the STEP Shorthand (**right**, current study). Assessments are shown for four independent raters (yellow = Rater 1, red = Rater 2, green = Rater 3, white = Rater 4). The STEP Shorthand demonstrates a narrower dispersion of values across raters, suggesting higher consistency across raters [7,8].

A strong correlation between chronological age and skeletal age was found for all three shorthands and for all four raters (Table 4) (Figure 2).

The ICC was rated as having an “excellent” agreement for all three shorthands, but the highest ICC was registered for the present shorthand (Table 5).

**Figure 3:**
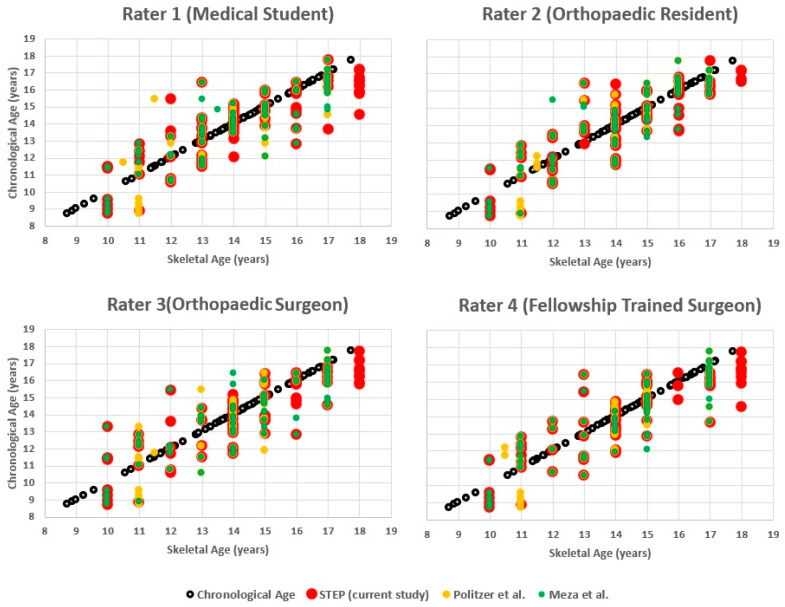
Chronological age versus skeletal age estimated with three shorthand methods (Politzer, Meza, STEP) by four independent raters with different levels of expertise. White circles indicate chronological age, while colored markers represent skeletal age estimations (yellow = Politzer, green = Meza, red = STEP) [7,8].

Moreover, the Cohen’s kappa for the intra-rater reliability showed a “strong” agreement in five comparisons of raters and a “moderate” agreement in two comparisons for both the present shorthand and for the Politzer et al. shorthand. Regarding the Meza et al. [7] shorthand, four comparisons or raters showed “strong” agreement, two “moderate” agreement, and one “good” agreement (Table 6 and Table 7) (Figure 3).

## 4. Discussion

The most important finding of the present study was that the Sagittal Tibial Epi-Physis (STEP) Shorthand provides a pragmatic and reliable MRI-based method for skeletal age assessment in skeletally immature patients with ACL injuries. The method demonstrated excellent inter-rater reliability (ICC 0.9436) and an equally strong correlation with chronological age in comparison with established atlases (rho 0.828–0.863, *p* < 0.001).

The STEP Shorthand’s clinical advantage lies in its pragmatic approach compared to existing MRI methods. While current techniques require time-consuming multi-planar evaluation of the knee, the STEP method focuses exclusively on proximal tibial physis morphology in a single sagittal slice. This targeted approach eliminates the need to assess secondary structures like the fibular head or interpret complex radiographic signs such as the “Oreo” configuration, which may increase interpretive complexity and require additional image planes or sequences.

Several key features enhance the method’s clinical implementation. The use of standard T1-weighted sequences, already integral to routine knee MRI protocols, means no additional imaging is required. The straightforward interpretive criteria demonstrated consistent reliability across evaluators with varying experience levels, from trainees to senior surgeons. Most importantly, by providing direct visualization of tibial physeal status at the actual surgical site, the method yields immediately useful data for clinical decision-making [13]. This is particularly valuable when determining whether physeal sparing or transphyseal reconstruction techniques are most appropriate for each patient [2,14].

Compared to the traditional Greulich and Pyle assessment, the STEP Shorthand offers a pragmatic knee-specific alternative. It completely eliminates the need for additional radiation exposure from hand–wrist radiographs while providing knee-specific skeletal maturity data. This focus allows surgical planning to be precisely tailored to each patient’s actual knee development status, which may help inform surgical planning and potentially reduce the risk of iatrogenic growth disturbance.

The clinical applications of this method could extend well beyond ACL reconstruction. Similar needs for accurate growth assessment exist in managing other pediatric knee conditions, including meniscal repairs, osteochondritis dissecans, and various physeal-sparing procedures [15].

The consistently high inter-rater reliability observed in our study suggests that the STEP Shorthand can be reliably implemented across diverse clinical settings and effectively incorporated into orthopedic training programs. Notably, its validation by raters with varying levels of experience and expertise supports its suitability for use by clinicians at all stages of their professional development. This standardization could help improve consistency in surgical decision-making for skeletally immature patients across institutions.

The method’s practical advantages could be relevant in today’s clinical environment, where efficiency and reliability are paramount. By simplifying the assessment process while maintaining accuracy, the STEP Shorthand addresses a practical need in pediatric orthopedic practice. Its capacity to deliver rapid, reproducible assessments without additional imaging makes it potentially suitable for busy clinical settings, enabling treatment decisions to be made at the first consultation without the need for further diagnostic exams.

Several limitations should be acknowledged. The study cohort lacked representation of patients younger than 9 years, and the female subgroup was relatively small, potentially limiting the generalizability of age-specific and sex-specific conclusions. It should nonetheless be acknowledged that the study cohort reflects a consecutive series of patients managed by a single surgeon at a high-volume referral center, where a substantial number of procedures are routinely performed. Therefore, this population can be regarded as consistent with the typical population of skeletally immature patients sustaining ACL injuries, reflecting the clinical spectrum typically encountered at high-volume referral centers.

Nonetheless, the single-institution nature of the data should be taken into account when interpreting the generalizability of the findings.

A direct validation of the atlas against hand radiographs was not undertaken. Howbeit, the development of the STEP Shorthand was grounded in the Pennock assessment method, which has already been validated using hand radiographs [8]. On this basis, it was deemed unnecessary to expose the patients included in the present study to an additional procedure such as hand radiography.

Future research should focus on validating the STEP Shorthand across more diverse patient populations, including younger children and those with atypical skeletal development patterns and other clinical conditions.

## 5. Conclusions

The STEP Shorthand represents a pragmatic and anatomically focused approach for MRI-based skeletal age assessment in skeletally immature patients with ACL injuries. By relying on a single sagittal T1-weighted image of the proximal tibial physis, the method is designed to facilitate feasibility and inter-rater reliability in routine clinical settings.

The incorporation of secondary features, aligned with those described in the established Pennock method, may contribute to the robustness of the STEP Shorthand. These additional markers can support skeletal age assessment, particularly in challenging or equivocal cases, without increasing interpretive complexity.

The present findings suggest that STEP demonstrates reliability characteristics comparable to existing MRI-based shorthands. Further external validation in larger, multicenter cohorts and in non-ACL populations is needed for a broader clinical implementation.

Future studies should also explore comparisons with established radiographic skeletal age methods and assess the added value of incorporating secondary MRI features in equivocal cases.

## Figures and Tables

**Table 1:** Bone age according to the features of the Sagittal Tibial Epi-Physis (STEP) Shorthand.

	Bone Age	
Features	Females	Males
Tibial apophysis without extension of epiphysis.	8 years	10 years
Tibial apophysis with extension of epiphysis.	9 years	11 years
Tibial apophysis with the presence of distal ossification center.	10 years	12 years
Tibial apophysis with partial fusion to the epiphysis and with “crack” line.	11 years	13 years
Tibial apophysis completely closed and tibial growth cartilage completely present, with “thick” thickness > 1.5 mm and without multilaminar appearance. As a secondary feature, the “Oreo” sign is still visible.	12 years	14 years
Tibial apophysis completely closed and tibial growth cartilage completely present, with “thin” thickness < 1.5 mm and without multilaminar appearance. As a secondary feature, the “Oreo” sign is not visible.	13 years	15 years
Tibial growth cartilage is closing only in the central portion, with femoral growth cartilage still completely visible. As a secondary feature, the status is confirmed on coronal slices.	14 years	16 years
Tibial growth cartilage is closed in the anterior and central portion, while femoral growth cartilage is closing only in its central portion. As a secondary feature, the status is confirmed on coronal slices.	15 years	17 years
Tibial growth cartilage is (almost) completely closed, while femoral growth cartilage closed in its anterior and central portions. As a secondary feature, the status is confirmed on coronal slices.	16 years	18 years

**Table 2:** Comparison of MRI features included in each shorthand.

	Males			Females		
	Shorthands			Shorthands		
MRI Features	Politzer et al. [8]	Meza et al. [7]	Present	Politzer et al. [8]	Meza et al. [7]	Present
Tibial apophysis extension of epiphysis	11 y	10 y	11 y	9 y	-	9 y
Patellar ossification 75–99%	-	11 y	-	-	-	-
Tibial apophysis ossification	12 y	12 y	12 y	10 y	10 y	10 y
Tibial apophysis partial fusion “crack”	13 y	13 y	13 y	10.5 y	11 y	11 y
Tibial apophysis ossified and fused (+“Oreo sign” visible)	14 y	15 y	-	11.5 y	12 y	
Tibial continuous cartilage, >1.5 mm, not multilaminar *	-	-	14 y	-	-	12 y
Patellar ossification completed	-	14 y	-	-	-	-
Femoral “Oreo sign” complete disappearance	15 y	-	-	13 y	-	-
Tibial continuous cartilage, <1.5 mm, not multilaminar *	-	-	15 y	-	-	13 y
Fibular styloid ossified	-	-	-	-	13 y	-
Tibial physis central closure	16 y	16 y	16 y	14 y	14 y	14 y
Fibular physis central closure	-	17 y	-	-	15 y	-
Femural physis central closure	17 y	-	-	15 y	-	-
Tibial physis anterior and central closure *	-	-	17 y	-	-	15 y
Tibial physis complete closure	-	-	18 y	-	16 y	16 y
Fibular physis complete closure	-	-	-	-	17 y	-

* Features not included in the original Pennock atlas.

**Table 3:** Median age at the presentation of the different MRI features in the Pennock atlas and in the present study.

	Males (n = 103)						Females (n = 27)					
MRI Feature	Age Ref.	Median Age (years)				Patient n°	Age Ref.	Median Age (years)				Patient n°
Tibial apophysis without extension of epiphysis	<11	**Pennock et al. [** 6 **]**	**-**	**Present Study**	9.4	12	<9	**Pennock et al. [** 6 **]**	**-**	**Present Study**	-	0
Tibial apophysis with extension of epiphysis	11		10.2		11.2	6	9		7.3		-	0
Tibial apophysis ossification center	12		11.8		12.3	6	10		10.1		-	0
Tibial apophysis partial fusion “crack”	13		12.8		13.1	6	11		10.7		-	0
Tibial continuous cartilage, >1.5 mm, not multilaminar ^a^	14		13.5		14.1	20	12		11.5		11.7	1
Tibial continuous cartilage, <1.5 mm, not multilaminar ^b^	15		15.6		15.1	18	13		13.9		12.8	5
Tibial physis central closure, Femoral physis fully present	16		16.1		15.9	13	14		14.3		14.1	7
Tibial physis anterior and central closure, Femoral physis central closure ^c^	17		16.9		16.7	17	15		15.1		14.8	7
Tibial physis complete closure, Femoral physis anterior and central closure ^d^	18		18.0		17.9	5	16		16.2		15.8	7

^a^ “Tibial apophysis ossified and fused + Oreo sign visible” in Pennock et al.; ^b^ “Femoral Oreo sign complete disappearance” in Pennock et al.; ^c^ “Femural physis central closure” in Pennock et al.; ^d^ “Tibial physis complete closure” in Pennock et al.

**Table 4:** Mean age according to the different shorthands.

Rater	STEP Shorthand (Current Study) ^a^	Politzer et al. [8] Shorthand ^a^	Meza et al. [7] Shorthand ^a^
**Rater 1 (MS)**	14.2 ± 2.5 years	14.1 ± 2.0 years	14.1 ± 2.1 years
**Rater 2 (OR)**	14.1 ± 2.2 years	14.0 ± 1.8 years	14.0 ± 2.0 years
**Rater 3 (OS)**	14.2 ± 2.4 years	14.2 ± 2.0 years	14.3 ± 2.3 years
**Rater 4 (FT)**	14.2 ± 2.4 years	14.2 ± 2.1 years	14.2 ± 2.3 years

^a^ *p*-value > 0.05 at ANOVA test.

**Table 5:** Correlation coefficient between chronological age and skeletal age with the different shorthands.

Chronological Age vs. Skeletal Age	Rater 1 (Medical Student)		Rater 2 (Orthopedic Resident)		Rater 3 (Orthopedic Surgeon)		Rater 4 (Fellowship Trained Surgeon)	
	Rho Correlation Coefficient	*p*-Value	Rho Correlation Coefficient	*p*-Value	Rho Correlation Coefficient	*p*-Value	Rho Correlation Coefficient	*p*-Value
STEP Shorthand	0.835 (95% CI 0.752–0.892)	*p* < 0.001	0.840 (95% CI 0.759–0.896)	*p* < 0.001	0.854 (95% CI 0.780–0.904)	*p* > 0.001	0.854 (95% CI 0.777–0.906)	*p* < 0.001
Meza et al. [7] Shorthand	0.848 (95% CI 0.770–0.901)	*p* < 0.001	0.828 (95% CI 0.741–0.887)	*p* < 0.001	0.829 (95% CI 0.744–0.887)	*p* > 0.001	0.836 (95% CI 0.751–0.894)	*p* < 0.001
Politzer et al. [8] Shorthand	0.843 (95% CI 0.763–0.897)	*p* < 0.001	0.857 (95% CI 0.783–0.907)	*p* < 0.001	0.863 (95% CI 0.793–0.910)	*p* > 0.001	0.848 (95% CI 0.769–0.902)	*p* < 0.001

**Table 6:** Interclass correlation coefficient (ICC) of the three bone age shorthands.

**STEP Shorthand (Current Study)**	ICC = 0.9436	(95%CI 0.9200–0.9619)
**Meza Shorthand**	ICC = 0.9279	(95%CI 0.8982–0.9513)
**Politzer Shorthand**	ICC = 0.9251	(95%CI 0.8943–0.9491)

**Table 7:** Inter-rater agreement (kappa) between the different raters.

**STEP Shorthand (Current Study)**		**Rater 2 (OR)**	**Rater 3 (OS)**	**Rater 4 (FT)**
	**Rater 1 (MS)**	0.757 (SE 0.036)	0.870 (SE 0.026)	0.821 (SE 0.029)
	**Rater 2 (OR)**	-	0.780 (SE 0.035)	0.832 (SE 0.032)
	**Rater 3 (OS)**	-	-	0.818 (SE 0.030)
**Politzer et al. Shorthand**		**Rater 2 (OR)**	**Rater 3 (OS)**	**Rater 4 (FT)**
	**Rater 1 (MS)**	0.795 (SE 0.038)	0.848 (SE 0.032)	0.825 (SE 0.039)
	**Rater 2 (OR)**	-	0.813 (SE 0.036)	0.795 (SE 0.038)
	**Rater 3 (OS)**	-	-	0.851 (SE 0.035)
**Meza et al. Shorthand**		**Rater 2 (OR)**	**Rater 3 (OS)**	**Rater 4 (FT)**
	**Rater 1 (MS)**	0.677 (SE 0.048)	0.822 (SE 0.036)	0.826 (SE 0.036)
	**Rater 2 (OR)**	-	0.768 (SE 0.037)	0.733 (SE 0.039)
	**Rater 3 (OS)**	-	-	0.823 (SE 0.033)

## Data Availability

The data that support the findings of this study are available on request. The data are not publicly available due to privacy restrictions.
